# Self-reported suicidal behaviour among people living with disabilities: prevalence and associated factors from a cross-sectional nation-wide survey in Bangladesh

**DOI:** 10.1186/s40359-024-01699-5

**Published:** 2024-05-09

**Authors:** Kamrun Nahar Koly, Aniqua Anjum, Rasma Muzaffar, Teresa Pollard, Taslima Akter, Zakia Rahman, Helal Uddin Ahmed, Julian Eaton

**Affiliations:** 1 Health System and Population Studies Division, International Centre for Diarrhoeal Disease Research, Bangladesh (icddr,b), 1212 Mohakhali, Dhaka, Dhaka Bangladesh; 2 North South University, Dhaka, Bangladesh; 3 Guy’s and St. Thomas’s NHS Foundation Trust, London, UK; 4 Center for Disability in Development, Dhaka, Bangladesh; 5 Christian Blind Mission (CBM) Global, Dhaka, Bangladesh; 6 National Institute of Mental Health, Sher-E-Bangla Nagar, Dhaka, Bangladesh; 7 Centre for Global Mental Health, London School of Hygiene and Tropical Medicine, London, UK; 8 CBM Global Disability and Inclusion, Laudenbach, Germany

**Keywords:** Suicidal Behaviour, SBQ-R, Mental Health, Persons with disability, Bangladesh

## Abstract

**Background:**

Disability marginalises a large portion of Bangladesh’s population. Global pre- and post-pandemic research evidently states that, this group is more prone to develop mental health problems, which increases the risk of self-harm and suicide among them. It is crucial to comprehend and mitigate the mental health challenges among the people with disabilities which in turn can promote their greater participation in community, and in national socioeconomic development. However, currently there is limited information available, regarding the suicidal behaviour of this group in Bangladesh. Therefore, this study aimed to investigate the prevalence and contributing factors of suicidal behaviour among people with disabilities.

**Method:**

A cross-sectional survey was conducted during September and October 2022, among the participants who had selected disabilities, by using probability proportional to size sampling technique across all eight divisions of Bangladesh. A semi-structured questionnaire comprising information about sociodemographic, lifestyle, health; and Suicidal Behaviour Questionnaire-Revision (SBQ-R) was used. The association between the determinants and mental health outcome was investigated using the Chi-square test, and the contributing factors were investigated using the multiple binary logistic regression.

**Result:**

About 10.45% of the participants reported to have suicidal behaviour (e.g., suicidal ideation, attempts, completed suicide), considering the cut-off score as 7 for the SBQ-R in the study period. Approximately, 40% respondents mentioned suicidal ideation in their lifetime, whereas, 9.01% had suicidal ideation over the past 12 months. Additionally, 8.87% of the person with disabilities, mentioned about their suicidal intent to the family members, and 5.94% reported the likelihood of suicide in the future. Being female, having multiple disabilities, and not being connected with family and friends were found to be significantly associated with suicidal behaviour.

**Conclusion:**

This research demonstrates the significance of treating mental health issues and expanding accessibility to pre-existing services to lessen the impact of the limitations generated by disabilities. Policymakers can utilize this baseline findings to design large scale research and develop measures for suicide prevention, and management for at-risk groups.

**Supplementary Information:**

The online version contains supplementary material available at 10.1186/s40359-024-01699-5.

## Background

Mental health is one of the most significant public health concerns worldwide [1]. Unaddressed common mental health problems often tend to increase the risk of suicidal behaviour among populations [2]. Suicidal behaviour can take various forms and intensities, including suicidal ideation, suicidal attempts, and actual suicide. Suicide claims approximately 730,000 lives globally annually, and according to the World Health Organization (WHO), over 79% of these occur in low- and middle-income countries (LMICs) [3–5]. In Bangladesh, reportedly eight per 100,00 people die because of suicide, leading to a total of 10,000 suicidal deaths cumulatively annually [6–10]. However, the actual rate is believed to be higher than the reported rate; since in Bangladesh it is common for incidents to be classified as accidental death rather than suicide, due to stigma and to avoid postmortem social repercussions [11].

Evidence suggests that disability itself is a significant risk factor for suicidal ideation [12]. Worldwide, about 15% of the population experience some form of disability such as physical disability (upper limb, lower limb), visual, speech and hearing disability [13]. A number of past studies revealed, around 5.6–10% of the Bangladeshi population have at least one form of disability [7–9]. Compared to persons without disabilities, persons with disabilities experience poor health and mental health outcomes [14]. Some contributing factors are: less access to healthcare facilities, lower levels of education, limited social and economic participation, and higher poverty rates for this marginalised population [15]. Chronic stress due to limitation in daily activities, stigma, discrimination, isolation from wider society, physical and financial dependency make them more vulnerable to mental health conditions [16]. Stigma related to suicide and disability, as well as lack of appropriate services, prevent them from seeking professional help reflecting double burden for this underprivileged population [17]. During the COVID-19 pandemic, additional symptoms indicating mental health issues were experienced, that could increase the risk of suicide behaviour among the person with disabilities [18]. Evidence from Higher Income Countries show that, persons with disabilities experienced increased burden (20.7–30.8%) of suicidal thoughts than the persons without disabilities (4.1–8.3%) during the COVID-19 pandemic [19–21]. Suicidal thoughts and behaviour differ from person to person depending on their age, sex, overall health, frequency of stressors and previous suicidal attempts or thoughts [14]. Previous studies reported that these factors influence the risk of suicidal behaviour [14].

However, Bangladesh still lacks any kind of epidemiological study concerning suicidal behaviour among people with disabilities. Although suicidality among the general population has been previously examined in several studies [22–26]. Those studies mainly determined the suicidal behaviour among persons with certain functional limitations like disabilities due to chronic illnesses, multiple sclerosis, Huntington disease and intellectual disabilities [27]. There is only one study conducted in Bangladesh which reported the prevalence of suicidal ideation as 23.9% among persons with disabilities [28]. However, this particular study assessed suicidal ideation in few districts of Bangladesh and did not use any assessment tool. Hence, there is a large knowledge gap about suicidal behaviour. On the contrary, our study aimed to conduct a nationwide survey to assess the prevalence and determinants of suicidal behaviour among this cohort.

## Methods

### Study design and settings

A cross-sectional study was conducted among people with disabilities from the largest disability rights based non-governmental organization (NGO) in Bangladesh, the Center for Disability in Development (CDD). Additionally, CDD works jointly with more than 350 national and international organizations for people with disabilities (OPDs) and disability-specific organizations (DSOs) [29]. The socio-demographical profile of participants covered the ages of 18 and 60 years and from Bangladesh’s eight divisions (highest administrative units), namely Dhaka, Chattogram, Barisal, Sylhet, Mymensingh, Khulna, Rajshahi, and Rangpur where the major CDD beneficiaries were based. The study included participants with a variety of physical (upper and lower extremity), speech, hearing, and visual impairments. As of 2021, CDD along with its collaborative partners (OPDs and DSOs) supported around 31,546 people with disabilities. A sampling frame comprising list of CDD beneficiaries was compiled using probability proportional to size (PPS) sampling. The overall sample was distributed among the selected area of beneficiaries regarding the share of the total beneficiaries per division in the selected OPDs and DSOs (Table 1). In addition to including each of the eight divisions, sampling was employed to determine geographical areas within each division based on the locations of the OPDs and DSOs. Savar, Mirpur, and Khilgaon from Dhaka; Bashkhali, Rangunia, and Shahid Nagar from Chattogram; Kalapara from Barisal; Dhubaura from Mymensingh; Dinajpur, Rangpur, and Mithapukur from Rangpur; Bagerhat from Khulna; Bagha from Rajshahi; and Kulaura from Sylhet were included. This study excluded those with intellectual disabilities, unable to communicate meaningfully, pregnant women, those under the age of 18, and those above the age of 60.

**Table 1:** Division-wise distribution of study participants

Division	Number of participants (N)	Percentage of participants from each divisions (%)
Dhaka	81	22.82
Chattogram	67	18.87
Sylhet	20	05.63
Barisal	20	05.63
Mymensingh	26	07.32
Khulna	33	09.3
Rajshahi	27	07.61
Rangpur	81	22.82
Total	355	

### Sample size

It was difficult to estimate the precise prevalence of suicidal behaviour due to the scarcity of research on the mental health problems that affect people with disabilities. Therefore, the prevalence found in research with Bangladeshi mothers who have child with autism spectrum disorder (ASD) was used to estimate the sample size for this study [30]. Suicidal behaviour is an outcome of the untreated common mental health issues, that is caused by social exclusion, barriers and poor quality of life among persons with disability. So, we considered the mothers as they are closely tied with their children with ASD and encounter similar type of obstacles in Bangladesh. Hence, the sample size was calculated using the formula below considering 15.7% prevalence of suicidal behaviour.

$$ n=\frac{{z}^{2}pq}{{d}^{2}}$$

where, *n* = number of samples; z = 1.96 (99% confidence level); p = prevalence estimate (15.7%); q = (1-p); and d = precision limit or proportion of sampling error 0.05.

Considering a 99% confidence interval and a 5% margin of error, a sample size estimate of 353 was calculated for this study. For this study, a larger sample size of 353 was considered based on the assumptions of a 10% non-response rate and a 1.5% design effect. Probability proportional to size (PPS) sampling technique was used to compute the sample size.

### Data collection procedure and measures

The data was collected using a semi-structured questionnaire developed in Bangla that included informed consent (see below). Based on the prior literatures, information regarding socio-demographic, lifestyle, health and disease, and healthcare seeking were included in the questionnaire [31–33]. During September and October 2022, the trained study team carried out face-to-face interviews to collect data. The local CDD staff enabled the team to locate the residences and contact the selected individuals from the sample list. The study participants were informed about the study objectives, the measures of protecting their anonymity and privacy of the participants. Written and verbal consents were collected from the participants and were given the choice to participate in the study. Additionally, all of participants had the flexibilities to have a caregiver who assisted them to answer during the most the interviews since it was mandatory for the person with visual, speech and hearing impairment. The research team was always accompanied by a trained CDD staff who knew sign language to support the person with the speech and hearing impairment. Some of the participants were also interviewed at the selected OPDs and DSOs to prioritize their preferences and compensated for their travel cost (100–200 BDT). The principal investigator and the co-investigators frequently checked the accuracy of the data collected. Following data collection, open responses were post-coded in accordance with the requirements. The post-coding was done based on previous literatures [31–33]. Participants with severe suicidal behaviour scores were given further referral to the collaborators for further mental health support.

### Measurements

To assess the primary outcome of suicidal behaviour, we considered the revised version of the Suicidal Behaviour Questionnaire-Revised (SBQ-R). A Bengali translated version was previously used among Bangladeshi university students during the second wave of COVID-19 pandemic and was also validated among the persons with autism, functional and motor disabilities in United States of America [34–36]. It is made up of four items, representing a different dimension of suicidality. SBQ-R item 1 into lifetime suicide ideation and suicide attempts; item 2 assesses the frequency of suicidal ideation over the past twelve months; item 3 indicates into the threat of suicidal behaviour; and item 4 evaluates self-reported likelihood of suicidal behaviour. Briefly, to be consistent with existing 4-item versions, the responses of the several items of the original questions were modified. We evaluated the SBQ-R total item scores separately, then calculated the overall response by combining the final scores. Based on previous literature, dichotomous responses were formed, considering 7 as cutoff [34, 36, 37].

Along with the previous literature and known confounders, we included the following socio-demographic information: lifestyle, health and disease, care-seeking behaviour related factors, to determine the association with suicidal behaviour [31–36]. Socio-demographic information were collected, related to age, gender, educational qualification, type of disability, occupation, religion, marital status, division, and area of residence. Regarding age and educational qualification, we considered completed years. Initially, the age was collected as a continuous variable. However, to understand the age specific suicidal behaviour, we categorized age into three different groups as 18–35 years, 36 to 54 years and above 54 years. In addition, the participants were asked about their food accessibility, sleeping duration, smoking habits, receipt of family and community support, and self-care practice, to understand lifestyle patterns. Moreover, sleep duration was reported as per their actual sleeping duration which were later categorized based on standard sleeping duration by the previous studies, i.e.,7 h. Furthermore, the health and disease-related section included history of non-communicable disease (NCDs) and other health-related issues. Additionally, the health care-seeking behaviour section included different relevant questions including their usual practice and type of barriers they faced while seeking healthcare.

### Statistical analysis

The study team entered, managed, cleaned, and processed all the data by using SPSS software version 26 and performed statistical analysis in SPSS software version 26 and STATA software version 13.0. Initially for descriptive statistics, both frequency and percentage were calculated. To identify the differences between the groups, we used the χ2 (Chi-square) test for categorical data and independent sample t-test for continuous data. We checked the linearity assumption between the factors and the outcome variable. We found there was a non-linear relationship between the factors and the outcome variable. Then we transformed the factors (age and educational qualification, sleeping hours, occupation) into categories. We estimated both unadjusted (crude) and adjusted odds ratio using simple and multiple logistic regression models considering different factors (age, gender, educational qualification, type of disability, occupation, religion, marital status, division, and area of residence) to see the effect of these factors on suicidal behaviour. Factors that were significant (with a p-value of less than 0.05) were considered for further estimation of the multiple logistic regression model. Assumptions of multiple logistic regression were checked, i.e., multicollinearity was checked (all included variables resulted in Variance Inflation Factor (VIF < 4), responses were independent, and responses were categorized and arranged in ascending order [38, 39].

## Results

### General characteristics of participants

A total of 355 participants were included in this study. The majority of the participants were 18–35 years old. Among the participants, fewer had education above secondary school (7.61%), higher nuclear families (77.75%),), a higher percentage of people were married (65.63%%) and 41.13% were urban residents. About 20.28% of the participants reported having multiple disabilities. Moreover, almost 45.07% of participants self-reported the presence of having NCDs. In addition, 64.31% of participants reported poor connectedness with family and friends, however, 65.63% reported that they received support from their community with their daily chores. However, 94.93% reported that they practice at least one form of self-care. Moreover, 54.93% of participants reported over-sleeping, and 72.68% stated that they faced problems with food accessibility (Table 1).

**Table 1:** Socio-demographic characteristic of the participants

Name of the variable		Frequency (n) Percentage (%) (sample)
**Gender**	Male	208 (58.59)
	Female	147 (41.41)
**Age**	Up to 35 years	170 (47.89)
	36 to 55 years	146 (41.13)
	56 years and above	39 (10.99)
**Education**	No formal education	106 (29.86)
	Up to higher secondary	222 (62.54)
	More than higher secondary	27 (7.61)
**Area of residence**	Urban	146 (41.13)
	Rural	209 (58.87)
**Religion**	Muslim	284 (80.0)
	Hindu	71 (20.0)
**Marital Status**	Unmarried	122 (34.37)
	Married	233 (65.63)
**Occupation**	Employed	155 (43.66)
	Unemployed	200 (56.34)
**Type of family**	Nuclear family	276 (77.75)
	Joint family	79 (22.25)
**Number of disabilities**	One	283 (79.72)
	Multiple	72 (20.28)
**Smoking behaviour**	No	183 (51.55)
	No, but former smoker	59 (16.62)
	Yes	113 (31.83)
**Non-communicable disease**	No	195 (54.93)
	Yes	160 (45.07)
**Connectedness with family and friends**	No	227 (64.31)
	Yes	126 (35.69)
**Self-care practice**	No	18 (5.07)
	Yes	337 (94.93)
**Support received from community members**	No	122 (34.37)
	Yes	233 (65.63)
**Sleeping duration**	Up to 7 h	160 (45.07)
	More than 7 h	195 (54.93)
**Food Accessibility**	No	97 (27.32)
	Yes	258 (72.68)
**Main earning member of the family**	Self	115 (32.39)
	Spouse	65 (18.31)
	Other family members	175 (49.30)

### Prevalence of suicidal behaviour among persons with disabilities

**Fig. 1:**
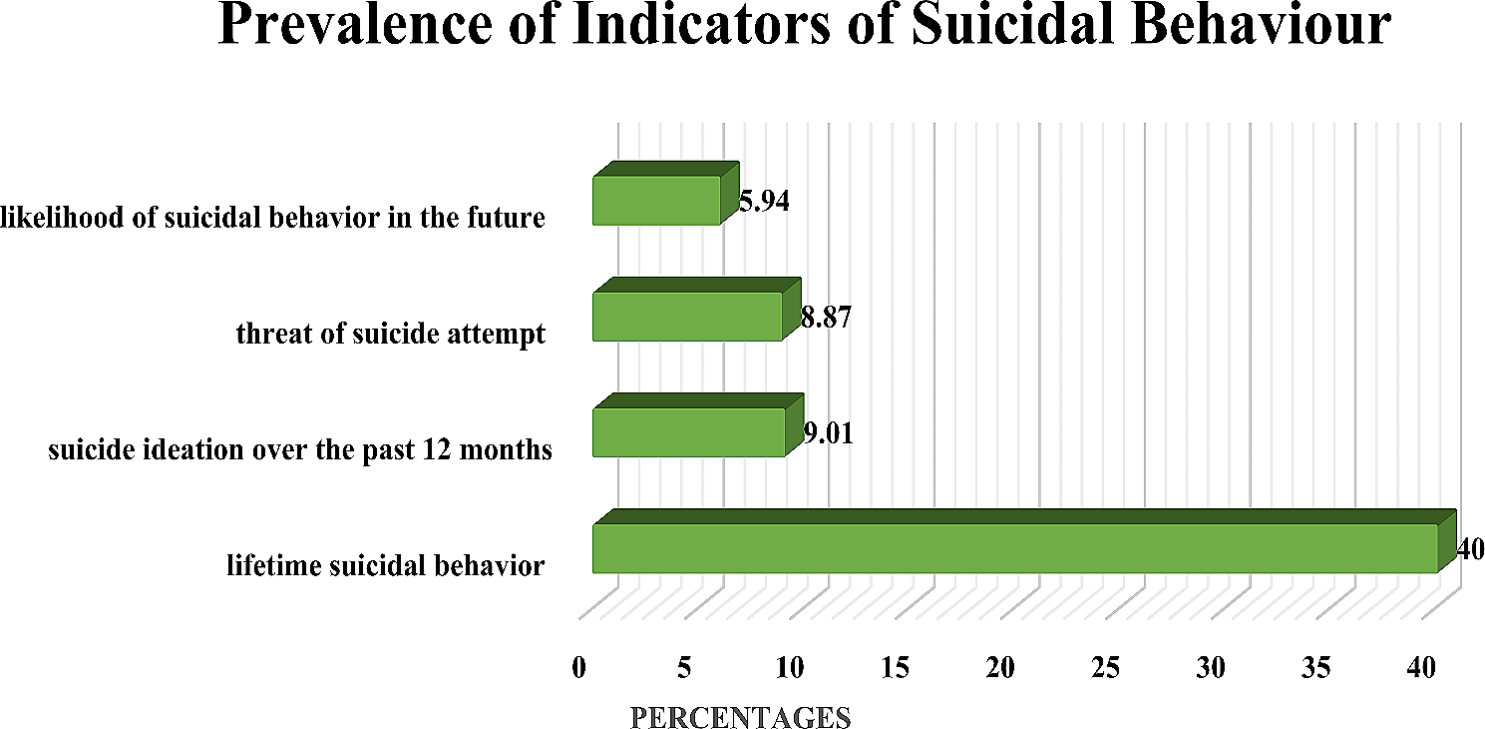
Prevalence of indicators of suicidal behaviour

The overall prevalence of suicidal behaviour among the persons with disabilities was 10.45%, considering the cut-off score as 7 for the SBQ-R. Whereas, about 40% reported having suicidal thoughts in their entire lifetime, 9.01% had suicidal ideation in the last 12 months, 8.87% threatened suicidal attempts and 5.94% reported a likelihood of suicidal behaviour in the future. ( Figure-1)

### Association between suicidal behaviour and other measures

Suicidal behavior was significantly associated with being female, having multiple disabilities, poor connection with family and friends, sleeping more than the standard duration, encountering problems in food accessibility (Table 2).

**Table 2:** Association between suicidal behavior and other measures

Name of the variable	Suicidal Behavior			Chi- Square *p*-value
	Absence of suicidal behavior	Presence of suicidal behavior		
**Gender**				4.104 (0.043)
Male	192 (92.31)		16 (7.69)	
Female	126 (85.62)		21 (14.38)	
**Age**				2.379 (0.304)
Up to 35 years	153 (90.53)		16 (9.47)	
36 to 55 years	127 (86.99)		19 (13.01)	
56 years and above	38 (94.87)		2 (5.13)	
**Education**				0.848 (0.655)
No formal education	95 (88.68)		12 (11.32)	
Up to higher secondary	200 (90.50)		21 (9.50)	
More than higher secondary	23 (85.19)		4 (14.81)	
**Area of residence**				2.798 (0.094)
Urban	126 (86.30)		20 (13.70)	
Rural	192 (91.83)		17 (8.17)	
**Religion**				0.0631 (0.802)
Muslim	255 (89.75)		29 (10.25)	
Hindu	63 (88.73)		8 (11.27)	
**Marital Status**				0.017 (0.897)
Unmarried	108 (89.26)		13 (10.74)	
Married	210 (89.70)		24 (10.30)	
**Occupation**				0.005 (0.944)
Employed	139 (89.68)		16 (10.32)	
Unemployed	179 (89.45)		21 (10.55)	
**Number of disabilities**				3.947 (0.047)
One	259 (91.17)		25 (8.83)	
Multiple	59 (83.10)		12 (16.90)	
**Type of family**				0.887 (0.346)
Nuclear family	244 (88.73)		31 (11.27)	
Joint family	74 (92.41)		6 (7.59)	
**Smoking behavior**				3.575 (0.167)
No	160 (86.89)		24 (13.11)	
No, but former smoker	52 (89.66)		6 (10.34)	
Yes	106 (93.81)		7 (6.19)	
**Non-communicable disease**				0.632 (0.427)
No	176 (90.72)		18 (9.28)	
Yes	142 (88.13)		19 (11.88)	
**Connectedness with family and friends**				4.586 (0.032)
No	197 (86.73)		30 (13.27)	
Yes	120 (94.44)		8 (5.56)	
**Self-care practice**				0.486 (0.486)
No	17 (94.44)		1 (5.56)	
Yes	301 (89.29)		36 (10.71)	
**Support received from community members**				1.508 (0.219)
No	105 (86.78)		16 (13.22)	
Yes	213 (90.99)		21 (9.01)32	
**Sleeping duration**				4.968 (0.026)
Up to 7 h	137 (85.53)		23 (14.47)	
More than 7 h	181 (92.82)		14 (7.18)	
**Food Accessibility**				9.691 (0.002)
No	79 (81.25)		18 (18.75)	
Yes	239 (92.64)		19 (7.36)	
**Main earning member of the family**				5.458 (0.065)
Self	105 (91.30)		10 (8.70)	
Spouse	53 (81.54)		12 (18.46)	
Other family members	160 (91.38)		15 (8.62)	

### Logistic regression analysis

From the crude model, female participants were 2.0 (CI: 1.0, 4.0) times more likely to have suicidal behaviour compared to males. Moreover, persons with multiple disabilities are 2.1 (0.9, 4.4) times more prone to have suicidal behaviour compared to persons with one disability. The odds of having suicidal behaviour for the persons who faced food inaccessibility were 2.9 (1.5, 5.8) times higher compared to those who did not faced any inaccessibility. Furthermore, odds were increased 0.5 (CI: 0.2, 0.9) times for longer sleepers. Additionally, participants who were not connected with their family and friends were found to have more than double the odds for having suicidal behaviour (OR: 2.6; CI: 1.1, 6.11).

A multiple binary logistic regression evaluated the associated factors of suicidal behaviour. The reference group was ‘no suicidal behaviour’. Being female (OR: 2.2; CI: 1.1, 4.9), faced problem in food accessibility (OR:2.6; CI: 1.3, 5.4), and not connected with their family and friends (OR: 2.9; CI: 1.2, 7.2) were significantly more likely to have suicidal behaviour (Table 3).

**Table 3:** Crude and adjusted logistic regression showing the associated factors of suicidal behaviour

Suicidal behaviour	COR [95% CI]	AOR [95% CI]
**Gender**		
Male (ref)	1	1
Female	2.016 [1.013, 4.013] *	2.355 [1.141, 4.863] *
**Number of disabilities**		
One (ref)	1	1
Multiple	2.099 [0.9970., 4.417] *	2.118 [0.964, 4.654]
**Food accessibility**		
Yes (ref)	1	1
No	2.903 [1.451, 5.807] **	2.641 [1.284, 5.432] **
**Sleeping duration**		
Up to 7 h (ref)	1	1
More than 7 h	0.457 [0.227, 0.921] *	0.502 [0.242, 1.043]
**Connectedness with family and friends**		
Yes (ref)	1	1
No	2.602 [1.108, 6.110] *	2.952 [1.21, 7.164] *

## Discussion

Suicide is a major social and public health issue which has been postulated to be influenced by the presence of a disability. Moreover, a number of studies have also significantly associated suicidal behaviour and suicides with common mental health conditions [40]. Importantly, the COVID-19 pandemic also led to deteriorating mental well-being of all populations, especially vulnerable populations like people with disabilities. However, very limited studies exploring suicidal behaviour among people with special needs exists, therefore our study assessed the prevalence and determinants of suicidal behaviour among persons with disabilities in Bangladesh. The findings may influence to create the scope for evidence-based and inclusive strategies for developing action plans to reduce the incident rates of suicide among people with disabilities.

Compared to HICs (High Income Countries), many studies have reported a higher suicide prevalence among populations of LMICs like Azerbaijan and Bangladesh, but very few of these studies highlighted the suicide prevalence among people with disabilities in LMICs [40]. Informing this gap, our study findings reported an overall prevalence of suicidal behaviour among the persons with disabilities to be 10.45%. Aligning with this, the few global studies available also depicted three folds increase of suicides among people with disabilities in comparison to people without disabilities [41]. Furthermore, as per the International Classification of Functioning, Disability and Health (ICF), the inter-connected factors like functional impairment, activity limitation and restricted participation, affect the way a person with disability can access and participate in society [42]. Such limitations and exclusion from society, also render people with disabilities at greater risk of common mental health conditions [42]. Moreover, unaddressed distress and mental health issues can lead to more severe mental health conditions, which are known to increase suicidality as well as being associated with shorter lifespan for a number of other reasons [40]. Hence, early interventions like early diagnosis, patient-profile based therapy, coordination between primary and secondary care sectors at individual, community and government level should be implemented.

As per prior studies, suicide is causally a heterogeneous phenomenon, varying with the patterns of risk factors across gender, age, culture, geographic location, and other person-specific factors like relationships, educational level, income level and so on [43]. As per a number of studies in LMICs, disability is already highly stigmatized in the societies [44, 45]. Hence, intersecting factors like having multiple disability and being females in the male dominant societies arise as additional challenges leading to suicidal behaviour [46–48]. Our study also reported suicidal behaviour to be significantly associated with factors like being female, having multiple disability, poor connection with family and friends, sleeping more than the standard duration, facing problems in food accessibility. Our findings were therefore coherent with existing research and theory.

Several international and national studies report a marked gender disparity in suicidal rates, with females being more prone to suicidal behaviour [49]. Although the rate of suicidal behaviour has been reported to be higher among women, the rate of successful suicide is higher among men in most global research [50]. Consistently, our study also reported being female as a significant factor contributing to suicidal behaviour. Similar to other LMICS like India and Pakistan, a patriarchal social system is dominant where women are subordinated to men both within the household and community levels [51]. In Bangladesh, women are the primary caregivers of the families and conversely considered as the economic and social burdens in their families. Owing to this, many females from impoverished families face child marriages, physical and sexual violence [49]. Moreover, the presence of disability-confounded with marital disharmony, perceived performance failure as wife, divorce based on infertility, and expulsion from the family, can instill suicidal behaviours among women with disabilities [52]. To overcome this disparity, gender-sensitive advocacy, and gender-specific mental health interventions are necessary.

Additionally,, our study findings emphasized that participants with multiple disabilities are more likely to have suicidal behaviour. Aligning with this, prior studies found that multiple disabilities increased the risk of suicidal thoughts and suicide by three to eight fold [53]. Moreover, compared to people with one disability, people with multiple disabilities are prone to increased health issues and more limitations in daily life activities, increasing their cumulative risk of being suicidal [48]. Hence, accommodations at family, community, policy, infrastructural levels and access to augmentative and alternative communication skills for health staff, leading to better access to social and health services for people with multiple disabilities is needed.

A number of prior studies across developed and resource-poor countries, reported low socio-economic status and food inaccessibility among people with disabilities are inter-related [54]. Following this, our study also stated respondents who faced food inaccessibility were more prone to suicidal behaviour. Unfortunately, few employment opportunities and the strong association between poverty and disability makes financial dependence for basic needs like food and shelter common among people with disabilities [55]. Therefore, inclusive and flexible policies to ensure access to poverty alleviation efforts, including livelihood and cash transfer programs, for person with disabilities should be ensured.

Furthermore, a plethora of research studies across the world report being connected with family and friends as a protective factor against suicidal behaviour [56]. However, many people with disabilities are deprived of their fundamental rights to participate in social and community life– for example enshrined in Article 19 of the Convention on the Rights of Persons with Disabilities– due to attitudinal barriers and stigma [57]. Additionally, family support is essential for people with disabilities to fulfill not only emotional needs, but resource provision or mobilization of supports and resources [58]. Our findings also confirmed poor connection with family and friends to be significantly associated with suicidal behaviour. Therefore, access to social rights and the exercise of community participation in the community should be encouraged.

Evidently, our study reported participants with insufficient sleeping hours are more inclined to suicidal behaviour. Likewise, previous research from LMICs highlighted that sleep disruptions, specifically insomnia symptoms and poor sleep quality significantly influence suicidal thoughts and suicide attempts [58]. To minimize this burden, this group should be educated to maintain a healthy life style including adequate sleeping time.

Suicidal behaviours often remain unreported or underreported and we recognise that our research only reflects the tip of the iceberg. Moreover, in a resource poor setting like Bangladesh, the burden of common mental health conditions is already higher among females, low income families and people with disabilities. Suicide reduction is an indicator for achieving the United Nations Sustainable Development Goals and a multisectoral strategy involving members from diverse sectors as well as the healthcare sector is necessary to prevent suicide among people with disabilities. As part of a national suicide prevention strategy for people with disabilities, regular media workshops at the national, regional, and local levels might be emphasized. Journalists can develop a self-regulating and self-monitoring system for the compassionate reporting of suicide cases. This research findings also have the potential to guide the formulation of additional suicide prevention interventions particularly for the people marginalised community.

### Strength and limitations

This was a cross-sectional study, no cause-and-effect relationships in between components were established. In order to investigate the potential risk of suicidal behaviour, longitudinal studies should be developed. Additionally, due to the lack of accessible communication resources, this study only included participants with physical, speech, hearing and visual impairment. Evidently, inclusion of psychosocial disabilities would have increased identified suicidal behaviour. Moreover, the cut-off points of these psychometric tools were established mostly among people without disability in Bangladesh context. Furthermore, suicidal behaviour is a complicated psychological phenomenon, which makes it difficult to accurately evaluate and categorize because it cannot be adequately captured by self-reported responses. However, due to self-reporting and related stigmas from positive responses, underreporting of suicide behaviours is probable. As people without impairments were not covered in our sample, it was not possible to contrast the suicidal behaviour of people with and without disabilities, which may have helped elucidate the relative impact of social factors vs. impairments and disability-specific issues.

Although during the consenting process, the trained field staff assured the participants of privacy, confidentiality and anonymity, there might be some possibilities of under reporting of the suicidal behaviour, since these issues are highly stigmatized in Bangladeshi.

To best of our knowledge, this is the first study to investigate the factors that may lead to suicidal behaviour among Bangladeshi people with disabilities. Since, it focused on the persons with disabilities, one of the most vulnerable population, who are largely understudied worldwide, these findings might be helpful for developing interventions programs for the susceptible group. Moreover, this was a population-based, large scale study which used previously validated instruments to assess suicidal behaviour. Furthermore, it covered almost all the covariates found significant in previous literatures. Moreover, the data were collected from the largest organization that works with people with disabilities, which followed a scientific approach for tracking them, which could be beneficial for designing large-scale studies.

## Conclusion

The COVID-19 pandemic has had drastic repercussions on mental health, especially among people with disabilities. With very limited research available on this specific population, we hope our nationwide study findings will establish the foundation for further research and interventions for people with disabilities. The findings identified the risk factors associated with suicidal behaviour among people with disabilities, and the study findings might help translate the into evidence-based interventions for a more inclusive mental health care system in Bangladesh. It is essential to ensure the nation’s disability support infrastructure is more aware of this issue, and that the mental health care system can provide better accessibility for people with disabilities.

### Electronic supplementary material

Below is the link to the electronic supplementary material.

Supplementary Material 1

## Data Availability

The datasets used and/or analysed during the current study are available from the corresponding author on reasonable request.
