# Supplementary material for: Self-reported suicidal behaviour among people living with disabilities: prevalence and associated factors from a cross-sectional nation-wide survey in Bangladesh
Source: BMC Psychol. 2024 May 9;12:231. doi: 10.1186/s40359-024-01699-5 (PMC11080185; doi:10.1186/s40359-024-01699-5)
Supplement: Supplementary file 1 — Supplementary Material 1 [file 40359_2024_1699_MOESM1_ESM.pdf]

Quantitative questionnaire for persons with disabilities (Bangla)

ID NO: ________________________
Date:____________________
Place:_________________________
Interviewer ID:
Phone Number of the Participant:

Study Title: Identifying common mental health issues and mental healthcare-seeking behavior among people with disability and inclusive mental health services in Bangladesh.			
Section A: Socio-demographic characteristics			
1	A1.	বয়স	অনুগ্রহপূর্বক বয়স---উল্লেখ করুন
2	A2.	লিঙ্গ	1. পুরুষ<br>2. মহিলা<br>3. অন্যান্য
3	A3.	শিক্ষাগত যোগ্যতা	1. প্রাতিষ্ঠানিক কোনো শিক্ষা নেই<br>2. প্রাথমিক শিক্ষা<br>3. মাধ্যমিক শিক্ষা<br>4. উচ্চ মাধ্যমিক শিক্ষা<br>5. (বিশ্ববিদ্যালয় অথবা উচ্চ)<br>6. অন্যান্য---
4	A3.	প্রতিবন্ধকতার ধরন-	1. শারীরিক বা অরথোপেডিক বৈকল্য<br>2. শ্রবণ প্রতিবন্ধকতা (বোবা অথবা কানে শোনে না)<br>3. বাক প্রতিবন্ধী<br>4. দৃষ্টি প্রতিবন্ধী (আংশিক অথবা পুরোপুরি)<br>5. মনোসামাজিক প্রতিবন্ধি/অক্ষমতা<br>6. শেখার অক্ষমতা

5	A5.	পেশা-	1. ছাত্র/ছাত্রী<br>2. গৃহিণী<br>3. সরকারি চাকুরীজীবী<br>4. বেসরকারি চাকুরীজীবী<br>5. ব্যবসা<br>6. অবসরপ্রাপ্ত<br>7. অন্যান্য<br><br>----------------------------------<br>(অনুগ্রহপূর্বক উল্লেখ করুন)
6	A6.	ধর্ম-	1. হিন্দু<br>2. মুসলিম<br>3.<br>3. অন্যান্য
7	A7.	বৈবাহিক অবস্থা-	1. অবিবাহিত<br>2. বিবাহিত<br>3. ডিভোরসি/বিধবা/আলাদা হয়ে গেছে
8	A8.	বিভাগ-	1. ঢাকা<br>2. চট্টগ্রাম<br>3. সিলেট<br>4. বরিশাল<br>5. ময়মনসিংহ<br>6. খুলনা<br>7. রাজশাহী<br>8. রংপুর
9	A9.	বসবাসের এলাকা-	1. শহর (স্থান----------------)<br>2. গ্রাম (স্থান---------------)<br>3. উপ-শহর (স্থান---------------)

10	A10.	পরিবারের ধরন-	1. একক পরিবার<br>2. যৌথ পরিবার
11	A11.	পরিবারের সদস্য সংখ্যা-	অনুগ্রহপূর্বক উল্লেখ করুন<br><br>------------------------------------
12	A12.	আপনাদের বাড়িতে কয়টা কক্ষ/ ঘর আছে?	অনুগ্রহপূর্বক উল্লেখ করুন<br><br>--------------------------------------
13	A13.	আপনাদের পরিবারের প্রধান উপার্জনকারী কে?	অনুগ্রহপূর্বক উল্লেখ করুন<br><br>---------------------------------------
14	A14.	পরিবারের মাসিক আয় (গড়)-	অনুগ্রহপূর্বক উল্লেখ করুন<br><br>---------------------------------------
	**Section B: Lifestyle related questions**		
15	B1.	আপনি কি খাবার নিয়মিত বা নিয়ম করে খান?	1. না<br>2. হ্যাঁ

16	B2.	যদি না হয়, তাহলে কেন নিয়মিত খান নি?	অনুগ্রহপূর্বক উল্লেখ করুন
17	B3.	আপনি প্রতিদিন গড়ে কত ঘন্টা ঘুমান?	1. <৭ ঘন্টা<br>2. ৭-৯ ঘন্টা<br>3. >৯ ঘন্টা
18	B4.	আপনি কি পান সুপারি / সিগারেট খান/ ধূমপান / করেন?	1. না<br>2. না, কিন্তু আগে খেতেন<br>3. হ্যাঁ
19	B5.	আপনার এই প্রতিবন্ধকতার কারনে পরিবারের সদস্যরা কি আপনাকে সহযোগীতা করে? (যেমন- যেকোন ধরনের অসুস্থতায় স্বাস্থ্যসেবা নেয়া, বা আর্থিক বা মানসিক বা চলাফেরায় বা অন্যান্য দৈনন্দিন কাজে)	1. না<br>2. হ্যাঁ<br>3. মোটামুটি
20	B6.	যদি হ্যাঁ হয়, তাহলে কি ধরনের সহযোগীতা করে থাকেন?	

21	B7.	আপনি কি আপনার এই প্রতিবন্ধকতার কারনে সমাজ থেকে যথেষ্ট সহযোগীতা পান? (যেমন-চলাফেরায়, বা স্বাস্থ্যকেন্দ্রে বা হাসপাতাল সেবাপ্রদানকারীদের থেকে বা অফিসে সহকর্মী বা স্কুলে সহপাঠীদের থেকে, কোন অনুষ্ঠান বা কোন প্রতিষ্ঠানে গেলে কোন সাহায্যের যেমন-চলাফেরায়, ইশারায় বুঝান/ সাইন লাঙ্গুয়েজ, শুনতে ও দেখতে কোন সাহায্যের প্রয়োজন হলে) ।	1. না<br>2. হ্যাঁ<br>3. মোটামুটি
22	B8.	আপনি কি নিজের শারীরিক যত্ন নেন?	1. না<br>2. হ্যাঁ
23	B9.	যদি হ্যাঁ হয়, তাহলে কিভাবে আপনি আপনার যত্ন নেন?	অনুগ্রহপূর্বক উল্লেখ করুন
24	B10<br>.	আপনি কি নিজের মানসিক যত্ন নেন?	1. না<br>2. হ্যাঁ

25	B11.	যদি হ্যাঁ হয়, তাহলে কিভাবে আপনি আপনার যত্ন নেন?	অনুগ্রহপূর্বক উল্লেখ করুন<br><br>--
26	B12.	আপনি অবসর সময় কিভাবে কাটান?	অনুগ্রহপূর্বক উল্লেখ করুন<br><br>--
	**Section C: Health and disease-related questions**		
27	C1.	আপনার কোন দীর্ঘস্থায়ী স্বাস্থ্য সমস্যা বা রোগ আছে?	1. না<br>2. হ্যাঁ

28	C2.	যদি উত্তর হ্যাঁ হয় তাহলে সমস্যা বা রোগগুলো উল্লেখ করুন?	1. ডায়াবেটিস<br>2. উচ্চরক্তচাপ<br>3. হৃদরোগ<br>4. হাঁপানি বা শ্বাসকষ্ট<br>5. কিডনীর রোগ<br>6. ক্যান্সার<br>7. গাইনোকোলজিক্যাল সমস্যা<br>8. মাসকিউলোস্কেলেটাল প্রবলেম<br>9. অন্যান্য<br><br>--------------------------------------(উল্লেখ করুন)
29	C3.	আপনি কতদিন ধরে এই রোগে বা স্বাস্থ্য সমস্যায় ভুগছেন?	অনুগ্রহপূর্বক উল্লেখ করুন<br><br>---
30	C4.	কোভিড-১৯ মহামারী আপনার এবং আপনার পরিবারের উপর কিধরনের প্রভাব ফেলেছে?	1. পরিবারের কোনো সদস্য বা আপনজনের মৃত্যু<br>2. নিজে আক্রান্ত হয়েছিলেন (যদি উত্তর ২ হয়, তাহলে নিম্নের প্রশ্নোগুলোর উত্তর দিন অন্যথায় সরাসরি C10 এ চলে যান।)<br>3. চাকরি হারিয়েছেন<br>4. অন্যান্য ক্ষতি (উল্লেখ করুন)_______________<br>5. না, কোন প্রভাব নেই

31	C5.	আপনি কোভিড-১৯ এ কতদিন আক্রান্ত ছিলেন?	অনুগ্রহপূর্বক উল্লেখ করুন<br><br>______________________
32	C6.	কোভিড-১৯ এ আক্রান্ত হবার পরে বর্তমানে এখনো কোন ধরনের প্রভাব রয়েছে কিনা?	অনুগ্রহপূর্বক উল্লেখ করুন<br><br>______________________
33	C7.	আপনি যদি কখনও কোভিড-১৯ দ্বারা আক্রান্ত হয়ে থাকেন তখন আপনি কোথায় চিকিৎসা নিয়েছিলেন?	1. বাড়িতে<br>2. হাসপাতালে<br>3. অন্যান্য (উল্লেখ করুন)<br><br>---
34	C8	আপনার কি করোনার সময় আপনার অন্য কোন স্বাস্থ্য সমস্যা হয়েছিল?	1. জ্বর<br>2. সর্দি<br>3. কাশি<br>4. শ্বাস কষ্ট<br>5. গলা ব্যথা<br>6. অন্যান্য

35	C9	আপনি কি কোভিড-১৯ টেস্ট করেছিলেন ?	1. না<br>2. হ্যাঁ
36	C10.	আপনি কি কোভিড-১৯ এর টিকা নিয়েছেন?	1. না<br>2. হ্যাঁ
37	C11.	যদি না নিয়া থাকেন তাহলে কেন নেন নি?	অনুগ্রহপূর্বক উল্লেখ করুন<br><br>--
38	C12.	কয়টি ডোজ নিয়েছেন?	1. একটি<br>2. দুইটি<br>3. তিনটি
	**Section D: Section D. Healthcare-seeking behaviour**		
39	D1.	গত ছয় মাসে/ ১ বছরে, আপনি আপনার কোন স্বাস্থ্য সমস্যার, উপসর্গ বা আঘাতের চিকিৎসার জন্য কোথাও গিয়েছিলেন?	1. না<br>2. হ্যাঁ

40	D2.	যদি না হয়, তাহলে কেন যান নি?	অনুগ্রহপূর্বক উল্লেখ করুন<br><br>---
41	D3.	যদি উত্তর হ্যাঁ হয়, তাহলে কোথায় গিয়েছিলেন?	1. কোথাও না<br>2. কবিরাজ<br>3. ধর্মীয় বা আধ্যাত্মিক স্থান<br>4. অ্যালোপ্যাথিক চিকিৎসক<br>5. হোমিওপাথিক প্র্যাকটিশনার<br>6. সরকারী হাসপাতাল<br>7. বেসরকারী হাসপাতাল বা ক্লিনিক<br>8. ওষুধের দোকান<br>9. অন্যান্য (উল্লেখ করুন)<br>---<br>---

42	D4.	আপনার বা আপনার পরিবারের কোন স্বাস্থ্য সংক্রান্ত সমস্যায় সাধারণত কোথায় যান?	1. কোথাও না<br>2. কবিরাজ<br>3. ধর্মীয় বা আধ্যাত্মিক স্থান<br>4. অ্যালোপ্যাথিক চিকিৎসক<br>5. হোমিওপাথিক প্র্যাকটিশনার<br>6. সরকারী হাসপাতাল<br>7. বেসরকারী হাসপাতাল বা ক্লিনিক<br>8. ওষুধের দোকান<br>9. অন্যান্য (উল্লেখ করুন)<br><br>---
43	D5.	আপনাকে সাধারনত কে বা কারা নিয়ে যায় এই সেবা গ্রহন করার জন্য?	অনুগ্রহপূর্বক উল্লেখ করুন<br><br>---

44	D6	আপনি আপনার চিকিৎসার খরচ কিভাবে জোগাড় করেন?	1. বর্তমান আয়<br>2. জমানো টাকা<br>3. ধার করে<br>4. সম্পত্তি বিক্রি করে<br>5. আত্মীয় সজনের সহায়তায়<br>6. অন্যান্য উৎস (উল্লেখ করুন)<br><br>--
45	D7.	স্বাস্থ্যসেবা নিতে গিয়ে আপনি কি কোন বাঁধার সম্মুখীন হয়েছেন?	1. না<br>2. হ্যাঁ
46	D8.	কি কি বাঁধার সম্মুখীন হয়েছেন?	1. কোথায় যেতে হবে না জানা<br>2. সহজলভ্য নয়<br>3. ব্যয়বহুল<br>4. সময়সাপেক্ষ<br>5. কুসংস্কার<br>6. ওষুধের পার্শ্বপ্রতিক্রিয়া<br>7. অন্যান্য (উল্লেখ করুন)<br><br>--

47	D9.	আপনার কি অতীতে কখনো এমন হয়েছে যে খুব মন খারাপ, ভয়, দুশ্চিন্তা বা ঘুম হত না ?	1. না<br>2. হ্যাঁ, যদি হ্যাঁ হয় কখন?<br>---------------------------<br>3. জানা নেই
48	D10.	এই একই সমস্যাগুলো আপনার পরিবারের সদস্যদের কারো মধ্যে ছিল বা আছে?	1. না<br>2. হ্যাঁ<br>3. জানা নেই
49	D11.	আপনি কি কখনো এসব সমস্যার জন্য / সমস্যার জন্য উপরোক্ত সমস্যার জন্য কোন ধরণের সহায়তা বা চিকিৎসাসেবা নিয়েছেন?	1. না<br>2. হ্যাঁ
50	D12.	যদি উত্তর হ্যাঁ হয়, তাহলে কোথায় গিয়েছিলেন?	1. কোথাও না<br>2. কবিরাজ<br>3. ধর্মীয় বা আধ্যাত্মিক স্থান<br>4. সাইকোলজিস্ট/ মনোবিদ<br>5. সাইকিয়াট্রিস্ট বা মনোরোগ বিশেষজ্ঞ<br>6. অন্যান্য (উল্লেখ করুন)<br>---

51	D13.	যদি না গিয়ে থাকেন তাহলে কেন যান নি?	(উল্লেখ করুন)<br><br>--

	**Section H: Assessment of suicidal behaviour (SBQ-R)**		
75	H1.	আপনার কি কখনো এমন লেগেছে যে বেচে থেকে কোন লাভ নেই, এই চিন্তা থেকে জীবনটা শেষ করে দেয়ার চেষ্টা করেছেন কখনো ?	1. কখনোই না<br>2. এটা শুধুমাত্র একটি ক্ষণস্থায়ী চিন্তা ছিল<br>3a. আমি অন্ততপক্ষে একবার আত্মহত্যার পরিকল্পনা করেছিলাম কিন্তু পরে আর চেষ্টা করি নি।<br>3b. আমি অন্ততপক্ষে একবার আত্মহত্যার পরিকল্পনা করেছিলাম এবং সত্যিকার অর্থেই মারা যেতে চেয়েছিলাম<br>4a. আমি আত্মহত্যা করার চেষ্টা করেছিলাম, কিন্তু মারা যেতে চাই নি<br>4b. আমি আত্মহত্যার চেষ্টা করেছিলাম, এবং সত্যিকার অর্থেই মারা যেতে চেয়েছিলাম

76	H2.	বিগত বছরে আপনি নিজেকে ঠিক কতবার আত্মহত্যার চেষ্টা করেছিলেন? (শুধু একটি টিক চিহ্ন দিন)	1. কখনোই না<br>2. কদাচিৎ (১ বার)<br>3. মাঝে মধ্যে (২ বার)<br>4. প্রায়ই (৩-৪ বার)<br>5. খুবই ঘন ঘন (৫ বার অথবা তার চেয়েও বেশি)
77	H3.	এই যে আপনার কি আত্মহত্যা চিন্তা হয়েছিল এই বিষয়ে আপনি কাউকে জানিয়েছেন ? (শুধু একটি টিক চিহ্ন দিন)	1. না<br><br>2a. হ্যাঁ, একবার, কিন্তু সত্যিকার অর্থে মারা যেতে চাই নি<br><br>2b. হ্যা, এক বার, এবং সত্যিকার অর্থেই মারা যেতে চেয়েছিলাম<br><br>3a. হ্যাঁ, একবারের চেয়ে বেশি, কিন্তু মারা যেতে চাইনি<br><br>3b. হ্যা, একবারের বেশি, এবং সত্যিকার অর্থেই মারা যেতে চেয়েছিলাম
78	H4.	এমন চিন্তা কখনো করেছেন ? আপনার কি কখনো আত্মহত্যার চেষ্টা করার সম্ভাবনা রয়েছে? (শুধু একটি টিক চিহ্ন দিন)	1. কখনোই না<br>2. কোনো সম্ভাবনা নেই<br>3. নিশ্চয়ই অসম্ভব<br>4. অসম্ভব<br>5. সম্ভবত<br>6. নিশ্চয়ই সম্ভাবনা আছে<br>7. খুবই সম্ভব

SBQ-R scoring:

The SBQ-R has 4-items, each tapping a different dimension of suicidality
- Item 1 taps into lifetime suicide ideation and/or suicide attempt.
- Item 2 assess the frequency of suicidal ideation over the past twelve months.
- Item 3 assess the threat of suicide attempt.
- Item 4 assesses the self-reported likelihood of suicidal behavior in the future.

Item 1 Scoring:
If 1 is selected = 1 point
If 2 is selected = 2 points
If 3 (3a) or 4 (3b) is selected = 3 points
If 5 (4a) or 6 (4b) is selected = 4 points

Item 2 Scoring:
If 0 is selected = 1 points
If 1 is selected = 2 points
If 2 is selected = 3 points
If 3 is selected = 4 points
If 4 is selected = 5 points

Item 3 Scoring:
If 1 is selected = 1 point
If 2 (2a) or 3 (2b) is selected = 2 points
If 4 (3a) or 5 (3b) is selected = 3 points

Item 4 Scoring:
If 0 is selected = 0 points
If 1 is selected = 1 points
If 2 is selected = 2 points
If 3 is selected = 3 points
If 4 is selected = 4 points
If 5 is selected = 5 points
If 6 is selected = 6 points

Total Score = Sum of items 1 through 4.
Possible scores 3-18

Quantitative questionnaire for persons with disabilities (English)

**Section A: Socio-demographic characteristics**		
A1.	Age	Please mention________ years
A2.	Gender	1. Male<br>2. Female<br>3. Others
A3.	Educational qualification	1.No formal education<br>2.Primary education<br>3.Secondary education<br>4.Higher secondary education<br>5.Tertiary (university or higher)
A4.	Type of disability-	
A5.	Occupation	1.Student<br>2.Housewife<br>3.Govt. employee<br>4.Private employee<br>5.Business<br>6.Retired<br>7.Others…………………………………………<br><br>(Please specify)
A6.	Religion-	1. Hindu<br>2. Muslim<br>3. Others

A7.	Division-	1. Dhaka<br>2. Chittagong<br>3. Sylhet<br>4. Barisal<br>5. Mymensingh<br>6. Khulna<br>7. Rajshahi<br>8. Rangpur
A8.	Area of residence-	1. Urban(location----)<br>2. Rural (Location--)<br>3. Semi-urban<br>9.
A9.	Marital status-	1. Unmarried<br>2. Married<br>3. Divorced/widowed/separated
A10.	Type of family-	1. Nuclear family<br>2. Joint family
A11.	Number of family members-	Please specify
A12.	How many rooms are there in your house?	Please specify
A13.	Who is the main earning member of your family?	Please mention
A14.	Monthly family income (average)-	Please specify<br>………………………………….. (BDT)
**Section B: Lifestyle related questions**		

B1.	Do you take your meals regularly?	1. No<br>2. Yes
B2.	How many hours do you sleep on average per day?	1. < 7 hours<br>2. 7-9 hours<br>3. > 9 hours
B3.	Do you smoke cigarette?	1. No<br>2. No, but former smoker<br>3. Yes
B4.	Are your family members supportive towards your disability related health condition? (E.g., taking health services in illness, financial or mental or to move or in other daily activities)	1. No<br>2. Yes<br>3. Moderately supportive
B7.	Do you get enough social supports from this disability perspective? (E.g., from healthcare providers in hospital or from colleagues in office or school classmates in school, or in other institutions or any event or social gatherings if you need any help to move, help with sign language/ visual aid/ hearing aid etc.)	1. No<br>2. Yes
B8.	Do you do a self-care?	1. No<br>2. Yes
B9.	If yes, how do you do your self-care?	Please mention----
**Section C: Health and disease-related questions**		
C1.	Do you have any underlying/previously diagnosed medical conditions?	1. No (If answer is No then please skip to C1)<br>2. Yes

C2.	If yes, mention your medical conditions?	1. Diabetes Mellitus<br>2. Hypertension<br>3. Heart Disease<br>4. Asthma/Respiratory Disease<br>5. Kidney Disease<br>6. Cancer<br>7. Others:<br><br>..............................(Please specify)
C3.	How long have you been diagnosed with/suffering from this medical condition?	Please mention<br><br>--
C4.	Did you have past history of any mental health issues?	1. No<br>2. Yes<br>3. Don't know
C5.	Do any of your family members have mental health issues:/ Do you have any family history of mental health issues?	1. No<br>2. Yes<br>3. Don't know
C6.	How has the COVID-19 pandemic affected you or your family?	1. Loss of any family member/s or close one/s<br>2. You got infected by COVID-19<br>3. Loss of job<br>4. Other financial loss<br><br>--<br>(Please specify)
C7.	If you ever got diagnosed with COVID-19, from where did you take the treatment?	1. Home<br>2. Hospital<br>Others (specify---)

C8.	Have you taken COVID-19 vaccine?	1. Yes<br>2. No
C9.	How many dose/s have you taken?	1. One dose<br>2. Two doses<br>3. Three doses
**Section D. Healthcare-seeking behaviour**		
D1.	In the last six months, did you go to attend the symptom or injury you have suffered from?	1. No<br>2. Yes
D2.	If yes, where did you go to attend the symptom or injury you have suffered from?	1. Public or private hospitals,<br>2. Primary care centers,<br>3. Independent health practitioners<br>4. Others<br>---<br>(Please mention-----)
D3.	Where do you usually visit/go for any health issues?	1. Don't visit<br>2. Kabiraj<br>3. Religious/ spiritual venue<br>4. Allopath practitioners<br>5. Homeopath practitioners<br>6. Govt. hospital,<br>7. Private hospital or clinic<br>8. Pharmacies or drug store<br>9. Others<br>--<br>(Please specify-----)
D4.	Did you ever seek help for your mental health issues?	1. No<br>2. Yes

D5.	If yes, where did you visit/go?	1. Don’t visit<br>2. Kabiraj<br>3. Religious/ spiritual venue<br>4. Psychologists<br>5. Psychiatrists<br>6. Others<br><br>---<br>(Please specify-----)
D6.	**Reasons for seeking care-**	1. Suggested by close kin<br>2. Believed to be effective<br>3. Easily accessible<br>4. Other (please specify)<br><br>---(Please specify)
D7.	If you did not visit, why?	Please mention
D8.	Sources of funds for managing treatment costs-<br>Current income<br>4<br>Other source	1. Current income<br>2. Savings<br>3. Loan<br>4. Selling assets<br>5. Relative's support<br>6. Other source
D9.	Total amount of money spent in treatment purpose-	Please specify
D10.	Did you face any challenges or barriers while seeking healthcare?	1. Yes<br>2. No

D11.	What are the barriers or challenges you face/ faced while seeking healthcare?	1. Don’t know where to seek help<br>2. Not accessible<br>3. Not affordable/ expensive<br>4. Hospitals are time consuming<br>5. Stigmatizing to visit hospital<br>6. Side effects of the medicine<br>7. Others<br><br>--<br>(Please specify)
**Section G: Assessment of suicidal behaviour (SBQ-R)**		
G1.	Have you ever thought about or attempted to kill yourself? (Check one only)	1. Never<br>2. It was just a brief passing thought<br>3a. I have had a plan at least once to kill myself but did not try to do it<br>3b. I have had a plan at least once to kill myself and really wanted to die<br>4a. I have attempted to kill myself, but did not want to die<br>4b. I have attempted to kill myself, and really hoped to die
G2.	How often have you thought about killing yourself in the past year? (Check one only)	1. Never<br>2. Rarely (1 time)<br>3. Sometimes (2 times)<br>4. Often (3-4 times)<br>5. Very Often (5 or more times)

G3.	Have you ever told someone that you were going to commit suicide, or that you might, do it? (Check one only)	1. No<br>2a. Yes, at one time, but did not really want to die<br>2b. Yes, at one time, and really wanted to die<br>3a. Yes, more than once, but did not want to do it<br>3b. Yes, more than once, and really wanted to do it
G4.	How likely is it that you will attempt suicide someday? (Check one only)	0. Never<br>1. No chance at all<br>2. Rather unlikely<br>3. Unlikely<br>4. Likely<br>5. Rather likely<br>6. Very likely

SBQ-R scoring:

The SBQ-R has 4-items, each tapping a different dimension of suicidality
• Item 1 taps into lifetime suicide ideation and/or suicide attempt.
• Item 2 assess the frequency of suicidal ideation over the past twelve months.
• Item 3 assess the threat of suicide attempt.
• Item 4 assesses the self-reported likelihood of suicidal behavior in the future.

Item 1 Scoring:
If 1 is selected = 1 point
If 2 is selected = 2 points
If 3 (3a) or 4 (3b) is selected = 3 points
If 5 (4a) or 6 (4b) is selected = 4 points

Item 2 Scoring:
If 0 is selected = 1 points
If 1 is selected = 2 points
If 2 is selected = 3 points
If 3 is selected = 4 points
If 4 is selected = 5 points

Item 3 Scoring:
If 1 is selected = 1 point
If 2 (2a) or 3 (2b) is selected = 2 points
If 4 (3a) or 5 (3b) is selected = 3 points

Item 4 Scoring:
If 0 is selected = 0 points
If 1 is selected = 1 points
If 2 is selected = 2 points
If 3 is selected = 3 points
If 4 is selected = 4 points
If 5 is selected = 5 points
If 6 is selected = 6 points

Total Score = Sum of items 1 through 4.
Possible scores 3-18
